# *In vivo* evaluation of radiotracers targeting the melanin-concentrating hormone receptor 1: [^11^C]SNAP-7941 and [^18^F]FE@SNAP reveal specific uptake in the ventricular system

**DOI:** 10.1038/s41598-017-08684-6

**Published:** 2017-08-14

**Authors:** Markus Zeilinger, Monika Dumanic, Florian Pichler, Lubos Budinsky, Wolfgang Wadsak, Katharina Pallitsch, Helmut Spreitzer, Rupert Lanzenberger, Marcus Hacker, Markus Mitterhauser, Cécile Philippe

**Affiliations:** 1 Department of Biomedical Imaging and Image-guided Therapy, Division of Nuclear Medicine, Medical University of Vienna, Vienna, Austria; 2 Department of Engineering, University of Applied Sciences Wiener Neustadt, Wiener Neustadt, Austria; 3 Department of Biomedical Imaging and Image-guided Therapy, Division of Molecular and Gender Imaging, Medical University of Vienna, Vienna, Austria; 4 Department of Inorganic Chemistry, University of Vienna, Vienna, Austria; 5 CBmed GmbH, Center for Biomarker Research in Medicine, Graz, Austria; 6 Department of Organic Chemistry, University of Vienna, Vienna, Austria; 7 Department of Pharmaceutical Chemistry, University of Vienna, Vienna, Austria; 8 Department of Psychiatry and Psychotherapy, Medical University of Vienna, Vienna, Austria; 9 Department of Pharmaceutical Technology and Biopharmaceutics, University of Vienna, Vienna, Austria; 10 Ludwig Boltzmann Institute for Applied Diagnostics, Vienna, Austria

## Abstract

The MCHR1 is involved in the regulation of energy homeostasis and changes of the expression are linked to a variety of associated diseases, such as diabetes and adiposity. The study aimed at the *in vitro* and *in vivo* evaluation of [^11^C]SNAP-7941 and [^18^F]FE@SNAP as potential PET-tracers for the MCHR1. Competitive binding studies with non-radioactive derivatives and small-animal PET/CT and MRI brain studies were performed under baseline conditions and tracer displacement with the unlabelled MCHR1 antagonist (±)-SNAP-7941. Binding studies evinced high binding affinity of the non-radioactive derivatives. Small-animal imaging of [^11^C]SNAP-7941 and [^18^F]FE@SNAP evinced high tracer uptake in MCHR1-rich regions of the ventricular system. Quantitative analysis depicted a significant tracer reduction after displacement with (±)-SNAP-7941. Due to the high binding affinity of the non-labelled derivatives and the high specific tracer uptake of [^11^C]SNAP-7941 and [^18^F]FE@SNAP, there is strong evidence that both radiotracers may serve as highly suitable agents for specific MCHR1 imaging.

## Introduction

**Figure 1:**
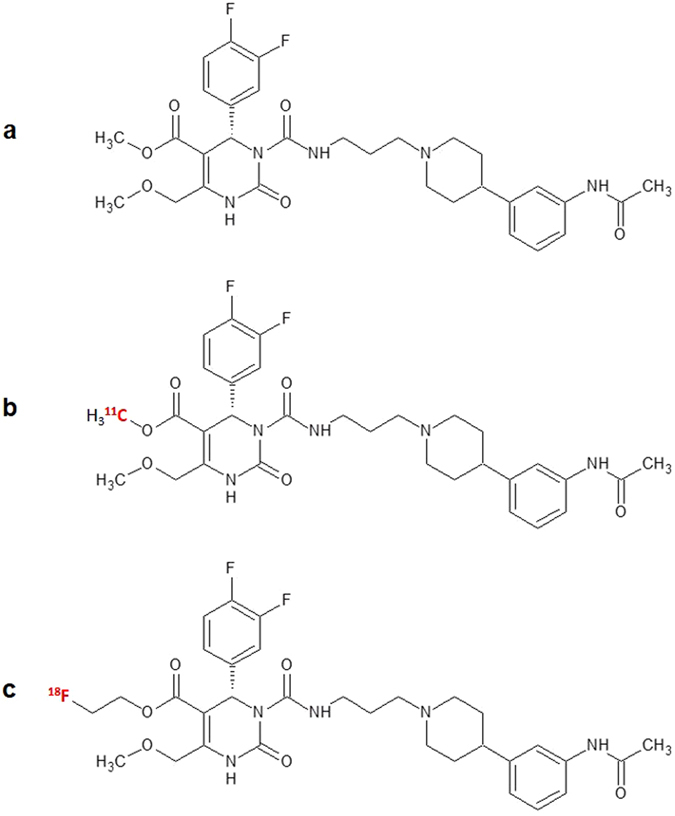
Chemical structures of the MCHR1 antagonists. Chemical Structures of (+)-SNAP-7941 (**a**), [^11^C]SNAP-7941 (**b**) and [^18^F]FE@SNAP **(c)**. The red colored atom indicates the position of the radioisotope introduced by radiolabeling.

The melanin-concentrating hormone (MCH) is a cyclic polypeptide consisting of 19 amino acids, produced predominantly by neurons in the lateral hypothalamus, incerto-hypothalamic area and zona incerta with extensive projections throughout the brain^1^. Besides, MCH is also found in peripheral organs and tissues, such as the pancreas^2^, colonic epithelial cells^3^ or adipocytes^4, 5^. The biological effects of MCH are mediated by two G-protein coupled receptors (GPCRs), termed MCH receptor 1 (MCHR1)^6–9^ and MCH receptor 2 (MCHR2)^10–13^. While the MCHR1 has been isolated from rodents and humans^6, 8^, the MCHR2 has thus far been identified in primates, dogs, ferrets and humans^10, 11^. Several lines of research evinced that MCH acts as an important mediator in the integrated regulation of energy homeostasis and body weight and is linked with diseases such as diabetes, insulin resistance, colitis and obesity^14–20^. The distribution of MCH and the expression of the MCHR1 in the brain outside of regions connected with nutritional behaviour, has led to the finding that MCH signalling is also involved in a variety of psychiatric disorders, such as depression and anxiety^21^. Previous studies have shown, that MCH acts as an important mediator of cerebrospinal fluid (CSF) homeostasis and positively controls cilia beat frequency of ependymal cells, whereas a lack of MCHR1 provokes an increase in ventricular size^22, 23^. Given the fact that ependymal cells and MCH neurons are both involved in glucose sensing^24–26^, MCH fibres could control the activity of ciliated cells to initiate an increase in CSF flow to meet metabolic needs. This strongly supports the idea that the MCH-system may also be involved in non-neuronal intercellular communication, but evidence is still lacking. However, to enable a quantitative *in vivo* assessment of the MCHR1 pharmacology and to facilitate preclinical to clinical translation, a suitable positron emission tomography (PET) tracer needs to be developed. Based on the specific MCHR1 antagonist (+)-methyl(4 S)-3-{[(3-{4-[3-(acetylamino)phenyl]-1piperidinyl}propyl)amino] carbonyl}-4-(3,4-difluorophenyl)-6-(methoxymethyl)-2-oxo-1,2,3,4-tetra-hydro-5-pyrimidenecarboxylate hydrochloride ((+)-SNAP-7941; Fig. 1a)^27^, the successful preparation of the first potential PET radiotracers [^11^C]SNAP-7941 (Fig. 1b) and the [^18^F]fluoroethylated analogue [^18^F]FE@SNAP (Fig. 1c) was performed^28, 29^. Since both tracers yield adequate amounts of radioactivity with suitable molar activity, initial preclinical evaluation has been accomplished^30, 31^.

Based on the preceding results, the current study focused on the quantitative *in vitro* and *in vivo* assessment of main biological and physicochemical properties of [^18^F]FE@SNAP and [^11^C]SNAP-7941 and the corresponding non-radioactive derivatives to enable confidence about the MCHR1 pharmacology. In detail, the aims of the study were (i) competitive binding studies with the non-radioactive derivatives, (ii) small-animal PET/CT and MRI studies of healthy rats with MCHR1 displacement and (iii) small-animal PET/CT and MRI studies of healthy rats under baseline conditions.

## Results

### Competitive binding studies

**Figure 2:**
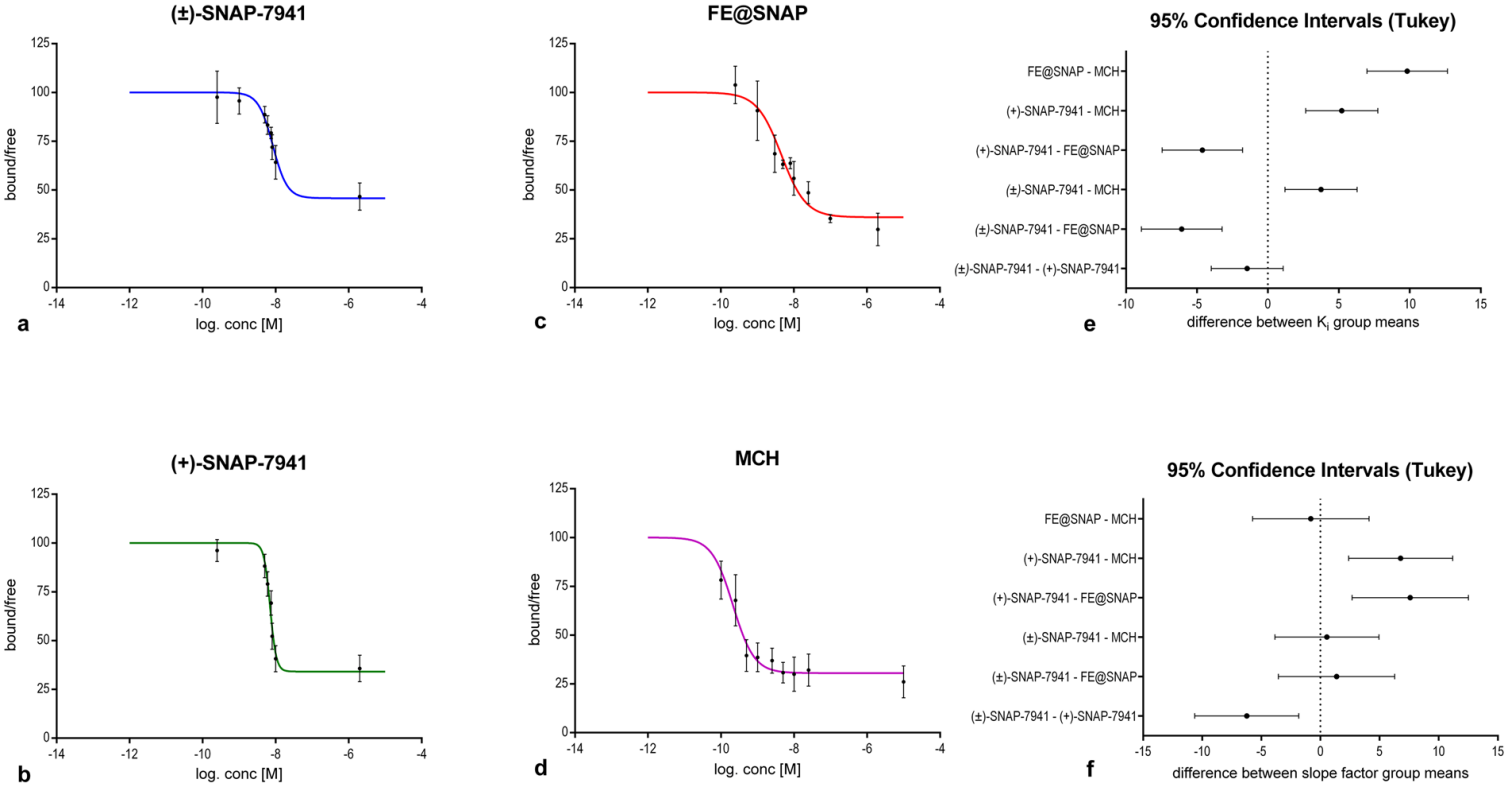
Competitive binding studies. Displacement of specific [^125^I]MCH binding on CHO-K1 cell membranes expressing the hMCHR1 in presence of different concentrations of (±)-SNAP-7941 (**a**), (+)-SNAP-7941 (**b**), FE@SNAP (**c**) and MCH (**d**). Differences between group means of the K_i_ (**e**) and the dedicated Hill slope factor (**f**) with corresponding 95% confidence intervals. Data are plotted as mean ± SEM from three independent experiments each performed in quadruplicate. If not visible the error bars are within the margin of the symbols.

Displacement of specific [^125^I]MCH binding on CHO-K1 membranes, expressing the hMCHR1, in presence of the different MCHR1 ligands evinced high binding affinity for (±)-SNAP-7941 and (+)-SNAP-7941, both in a low nanomolar range, whereas the binding affinity of FE@SNAP was determined to be significantly lower (Fig. 2 and Table 1). Differences of the K_i_ values between (±)-SNAP-7941and (+)-SNAP-7941 were found to be statistically not significant (*P* > 0.05). MCH revealed high binding affinity with a K_i_ in a low nanomolar range and was found to be in good agreement with previously reported values. Hill slope factors of (±)-SNAP-7941, FE@SNAP and MCH indicated no binding cooperativity and were determined to be statistically not significantly different (*P* > 0.05). On the contrary the Hill slope factor of (+)-SNAP-7941 was found to be significantly higher and revealed a strong positive cooperativity. A detailed summary of corresponding IC_50_, K_i_ and Hill slope factors of the dedicated MCHR1 ligands is shown in Table 1.

**Table 1:** Binding affinity of MCHR1 antagonists.

Compound	IC_50_ [nM]	K_i_ [nM]	Hill slope factor
(±)-SNAP-7941	5.61 ± 1.06	3.91 ± 0.74	2.66 ± 0.39
(+)-SNAP-7941	7.70 ± 0.35	5.37 ± 0.25	8.89 ± 1.59
FE@SNAP	14.32 ± 1.61	9.98 ± 1.12	1.30 ± 0.33
MCH	0.24 ± 0.05	0.16 ± 0.03	2.11 ± 0.63

Displacement of specific [^125^I]MCH binding on CHO-K1 cell membranes expressing the hMCHR1. Data in the table are expressed as mean ± SEM from three independent experiments each performed in quadruplicate.

### Small-animal imaging

**Figure 3:**
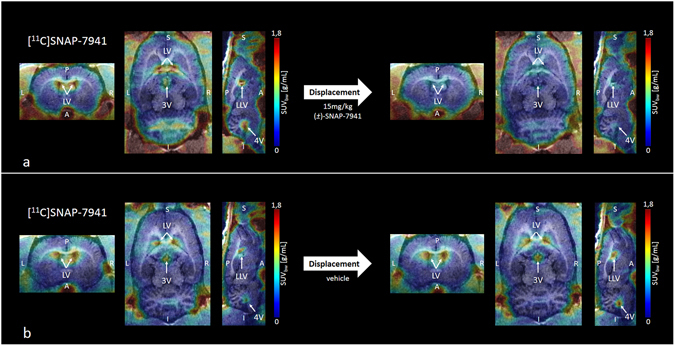
Small-animal PET/MR brain images of [^11^C]SNAP-7941. Exemplary small-animal PET/MR images of representative coronal (left), horizontal (centre) and sagittal (right) planes of a rat brain with [^11^C]SNAP-7941 before and after administration of 15 mg/kg (±)-SNAP-7941 (**a**) and with [^11^C]SNAP-7941 before and after administration of the corresponding vehicle (**b**). PET data are contributed to summation images from 0–15 minutes (before displacement) and from 15–45 minutes (after displacement). Anatomical structures are indicated by arrows (**LV** = lateral ventricle; **LLV** = left lateral ventricle; **3V** = third ventricle; **4V** = fourth ventricle).

**Figure 4:**
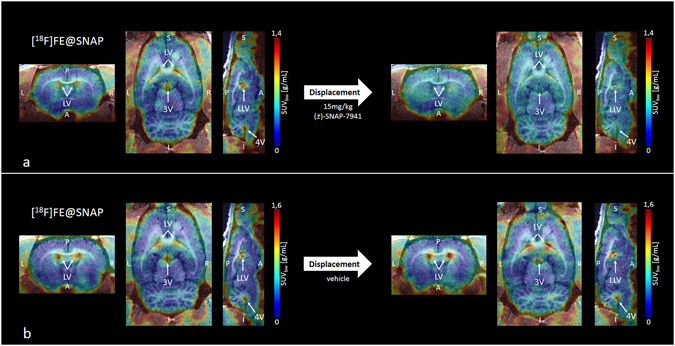
Small-animal PET/MR brain images of [^18^F]FE@SNAP. Exemplary small-animal PET/MR images of representative coronal (left), horizontal (centre) and sagittal (right) planes of a rat brain with [^18^F]FE@SNAP before and after administration of 15 mg/kg (±)-SNAP-7941 (**a**) and with [^18^F]FE@SNAP before and after administration of the corresponding vehicle (**b**). PET data are contributed to summation images from 0–20 minutes (before displacement) and from 20–60 minutes (after displacement). Anatomical structures are indicated by arrows (**LV** = lateral ventricle; **LLV** = left lateral ventricle; **3V** = third ventricle; **4V** = fourth ventricle).

**Figure 5:**
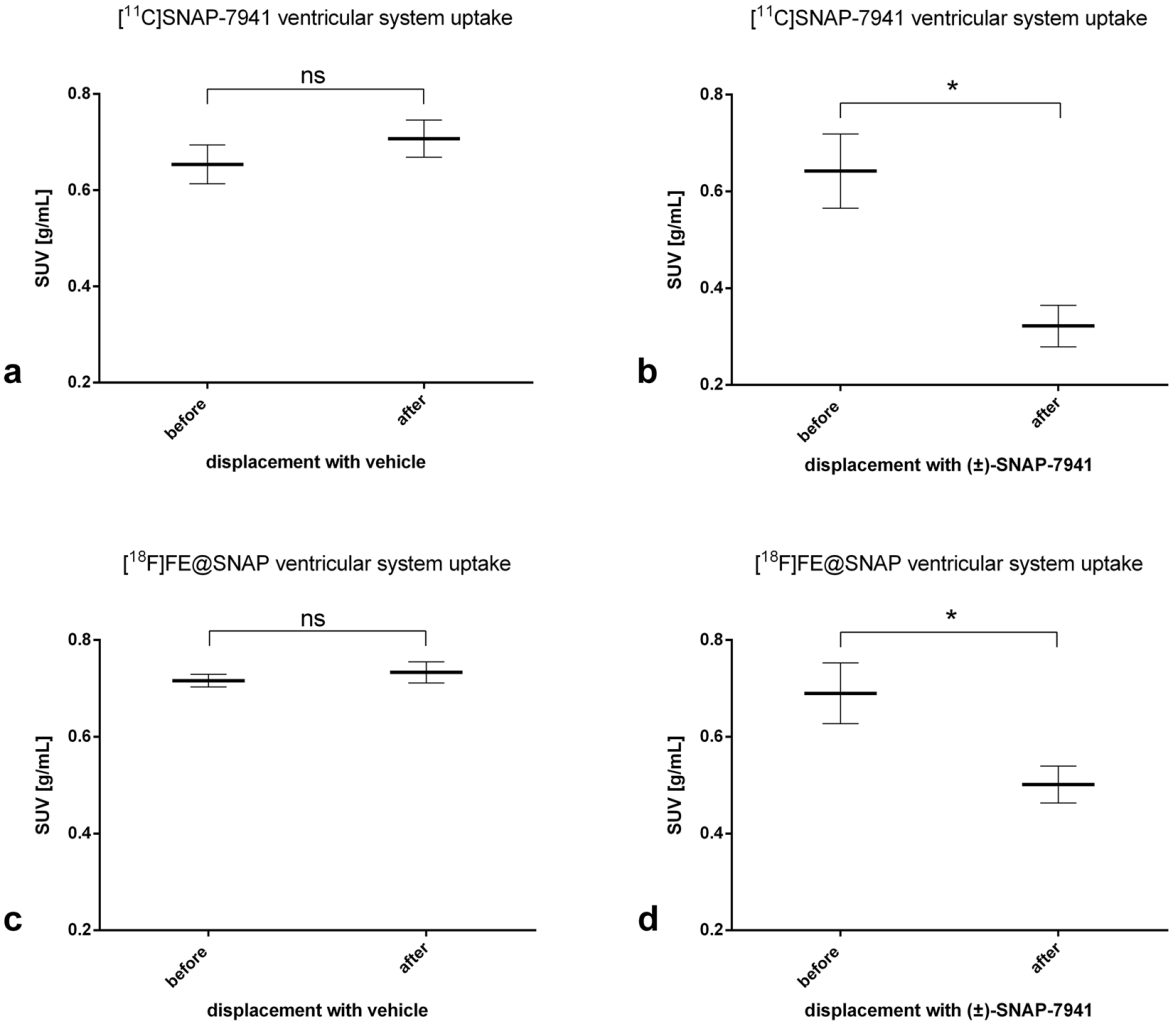
Radiotracer uptake in the ventricular system. Radiotracer uptake in the ventricular system of [^11^C]SNAP-7941 before and after displacement with either vehicle (**a**) or 15 mg/kg (±)-SNAP-7941 **(b)** and [^18^F]FE@SNAP before and after displacement with either vehicle (**c**) or 15 mg/kg (±)-SNAP-7941 (**d**). Data are displayed as mean ± SEM from independent experiments (n ≥ 3). Differences among groups were tested using a two-tailed parametric paired t-test (ns = P > 0.05; *P < 0.05). If not visible, error bars are within the margin of the symbols.

**Figure 6:**
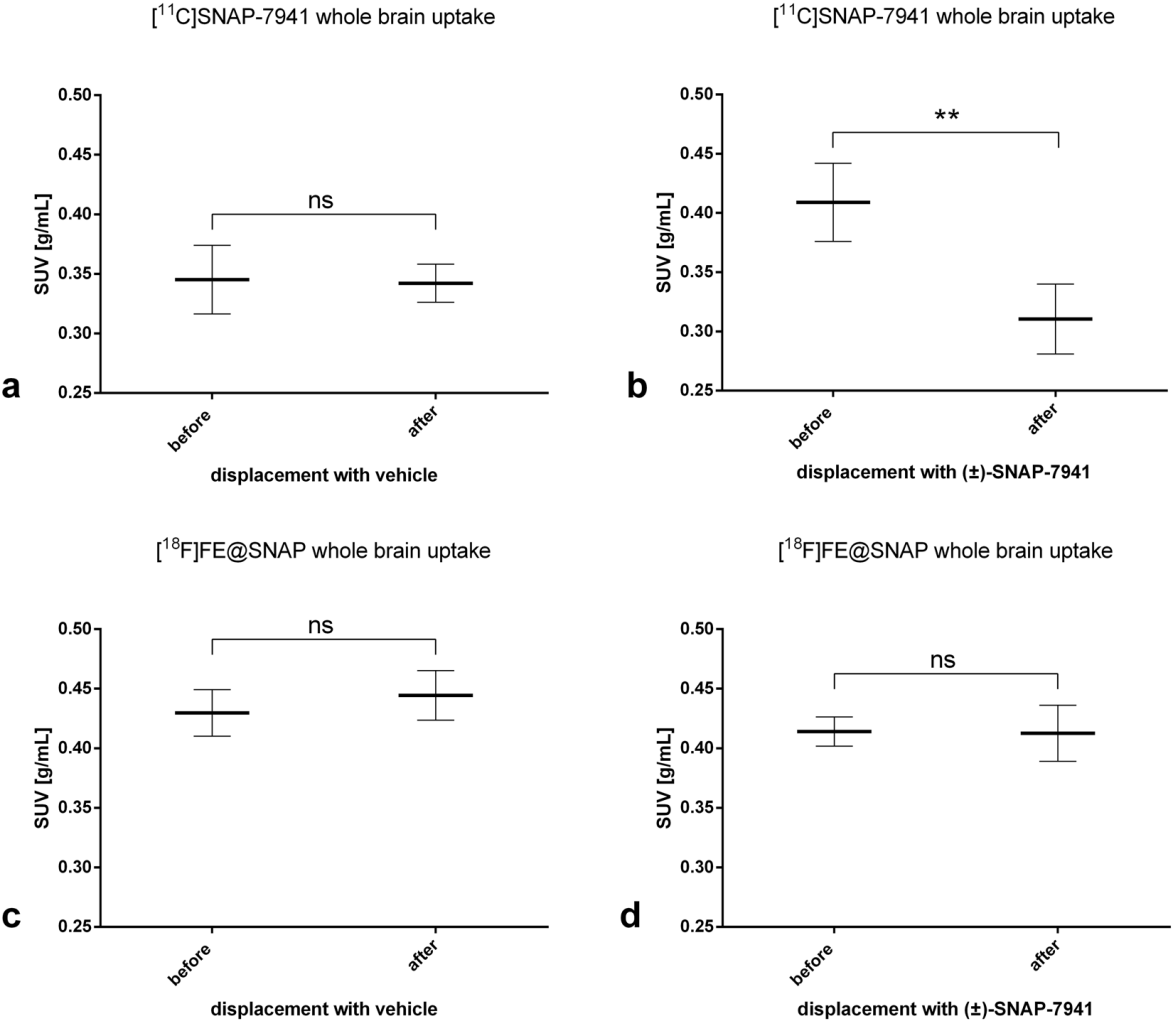
Radiotracer uptake in the whole brain. Whole brain uptake of [^11^C]SNAP-7941 before and after displacement with either vehicle (**a**) or 15 mg/kg (±)-SNAP-7941 (**b**) and [^18^F]FE@SNAP before and after displacement with either vehicle (**c**) or 15 mg/kg (±)-SNAP-7941 (**d**). Data are displayed as mean ± SEM from independent experiments (n ≥ 3). Differences among groups were tested using a two-tailed parametric paired t-test (ns = P > 0.05; **P < 0.01). If not visible, error bars are within the margin of the symbols.

**Figure 7:**
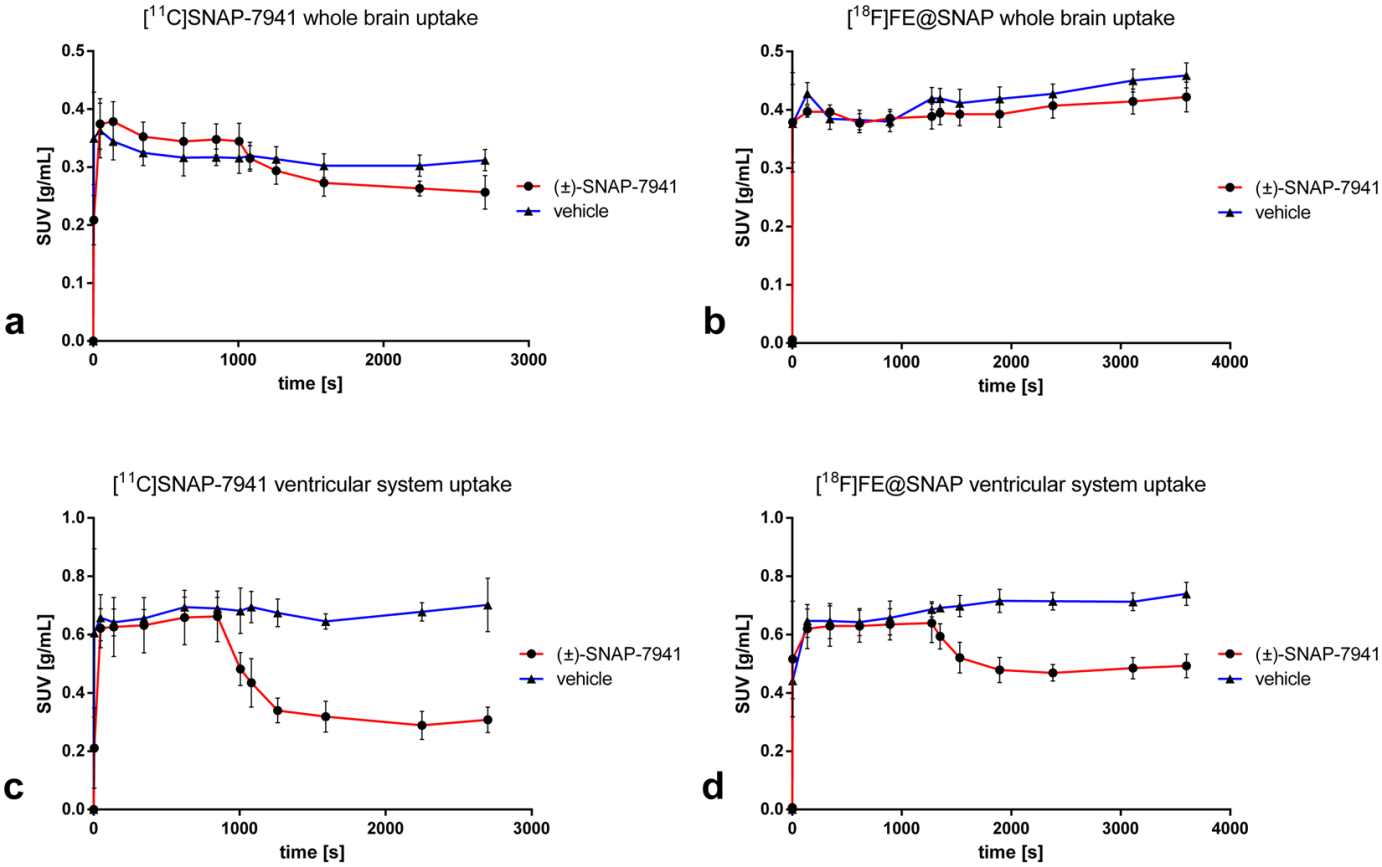
Time–activity curves of [^11^C]SNAP-7941 and [^18^F]FE@SNAP. Time–activity curves (mean SUV ± SEM) indicating the whole brain uptake of [^11^C]SNAP-7941 (**a**) and [^18^F]FE@SNAP (**b**) and the uptake in the ventricular system of [^11^C]SNAP-7941 (**c**) and [^18^F]FE@SNAP (**d**). Curves depict basal tracer kinetics followed by displacement with either 15 mg/kg (±)-SNAP-7941 (red line with circles) or the vehicle (blue line with triangles). Data are expressed as mean ± SEM from three independent experiments. If not visible, error bars are within the margin of the symbols.

Small-animal PET/MRI brain scans in healthy rats evinced high radiotracer uptake in the entire ventricular system for [^11^C]SNAP-7941 and [^18^F]FE@SNAP respectively, whereas the uptake in other brain regions was found to be comparable low. Representative triplanar PET/MRI rat brain scans are shown in Fig. 3 ([^11^C]SNAP-7941) and Fig. 4 ([^18^F]FE@SNAP). Differences between the mean SUV in the ventricular system before and after displacement with 15 mg/kg (±)-SNAP-7941 were found to be statistically significant for both [^11^C]SNAP-7941 (Figs 3a and 5b) and [^18^F]FE@SNAP (Figs 4a and 5d). As opposed to this no effect was determined after displacement of the basal radiotracer uptake with the vehicle for [^11^C]SNAP-7941 (Figs 3b and 5a) and [^18^F]FE@SNAP (Figs 4b and 5c). A detailed summary of all mean SUV values, of both radiolabelled compounds before and after displacement with (±)-SNAP-7941, the corresponding vehicle and corresponding *P* values are shown in Table 2. Whole brain uptake of [^11^C]SNAP-7941 was found to be significantly reduced after displacement with (±)-SNAP-7941 (Fig. 6b), whereas in the group where the vehicle was used as displacement agent differences were proved to be statistically not significant (Fig. 6a). In contrast to that, differences of the whole brain uptake of [^18^F]FE@SNAP before and after displacement were found to be statistically not significant for both displacement with (±)-SNAP-7941 (Fig. 6d) and the vehicle (Fig. 6c). Since the brains of all groups were counted in the Gamma Counter too, the findings of the micro PET scans could be confirmed. *Ex-vivo* brain uptake of [^11^C]SNAP-7941 measured in the Gamma Counter revealed a normalized value (mean ± SEM, expressed as %ID/g) of 0.023 ± 0.002 for the vehicle group and 0.015 ± 0.002 for the group where displacement was introduced via (±)-SNAP-7941. Differences of group means were proven to be statistically significant (*P* = 0.0156). *Ex-vivo* brain uptake of [^18^F]FE@SNAP revealed a normalized value (mean ± SEM, expressed as %ID/g) of 0.020 ± 0.001 for the vehicle group and 0.018 ± 0.003 for the group where displacement was introduced via (±)-SNAP-7941. No significant effect between both groups was observed (*P* = 0.4547). TACs of the whole brain and the ventricular system of [^11^C]SNAP-7941 and [^18^F]FE@SNAP before and after displacement with either the vehicle compound or with (±)-SNAP-7941 are shown in Fig. 7. [^11^C]SNAP-7941 displayed fast tracer equilibrium and a stable signal over the whole time course of the experiment for the group where the vehicle was used for displacement (Fig. 7a and c, blue line with triangles), whereas a clear drop in the TACs for both, whole brain and the ventricular system, was observed after displacement with 15 mg/kg (±)-SNAP-7941 (Fig. 7a and c, red line with circles). TACs of [^18^F]FE@SNAP evinced fast tracer equilibrium and a stable signal over the whole course of investigation for the whole brain and the ventricular system in the group where displacement was introduced via the vehicle (Fig. 7b and d, blue line with triangles). A clear drop of the TAC of [^18^F]FE@SNAP was shown immediately after displacement with 15 mg/kg (±)-SNAP-7941 (Fig. 7d, red line with circles), whereas no differences in the TAC of the whole brain was observed after treating the animals with the respective antagonist (Fig. 7c, red line with circles).

**Table 2:** Radiotracer uptake in the whole brain and the ventricular system.

[^11^C]SNAP-7941 displacement with (±)-SNAP-7941		SUV_bw_ [g/mL]	
*Region*	*Baseline*	*Displacement*	*P value*
Whole brain	0.41 ± 0.03	0.31 ± 0.03	0.003
Ventricular system	0.64 ± 0.08	0.32 ± 0.04	0.012
**[** ^**11**^ **C]SNAP-7941 displacement with vehicle**		**SUV**_**bw**_ **[g/mL]**	
Whole brain	0.35 ± 0.03	0.34 ± 0.02	0.845
Ventricular system	0.65 ± 0.04	0.71 ± 0.04	0.184
**[** ^**18**^ **F]FE@SNAP displacement with (±)-SNAP-7941**		**SUV**_**bw**_ **[g/mL]**	
Whole brain	0.41 ± 0.01	0.41 ± 0.02	0.936
Ventricular system	0.69 ± 0.06	0.50 ± 0.04	0.022
**[** ^**18**^ **F]FE@SNAP displacement with vehicle**		**SUV**_**bw**_ **[g/mL]**	
Whole brain	0.43 ± 0.02	0.44 ± 0.02	0.198
Ventricular system	0.72 ± 0.01	0.73 ± 0.02	0.292

SUV_bw_ mean values for the radiotracer uptake in the whole brain and the ventricular system of [^11^C]SNAP-7941 and [^18^F]FE@SNAP, both before and after displacement with either 15 mg/kg (±)-SNAP-7941 or vehicle with corresponding P values. Data in the table are expressed as mean ± SEM from independent experiments (n ≥ 3). Differences between baseline and displacement conditions were tested using a two-tailed parametric paired t-test. Values of P < 0.05 were considered as statistically significant.

## Discussion

The *in vivo* quantification of MCHR1 pharmacology is a crucial step for the better understanding of the pathogenesis of a variety of endocrine disorders like obesity, diabetes and insulin resistance. Therefore a specific PET radiotracer for MCHR1 imaging is of high scientific interest, since it comprises several advantages for clinical medicine and biomedical research, such as monitoring of the hormone receptor status and related pathologies *in-vivo*, compound dose selection and the *in vivo* quantification of the MCHR1 as a risk factor and early diagnostic tool for adiposity, diabetes and insulin resistance. To foster the *in-vivo* imaging of the MCHR1, PET radiotracer development was initiated^28–33^. Based on the preceding results, the current study focused on the quantitative *in vitro* and *in vivo* assessment of main biological and physicochemical properties of [^18^F]FE@SNAP and [^11^C]SNAP-7941 and corresponding non-radioactive derivatives to enable confidence about the MCHR1 pharmacology. Due to the low density of the MCHR1 in the human brain (B_Max_ = 5.8 ± 0.3 fmol/mg)^34^, a high binding affinity is mandatory. In the present paper we demonstrated high binding affinity in a low nanomolar range for our non-labeled reference compounds FE@SNAP, (±)-SNAP-7941 and (+)-SNAP-7941 in competition experiments using CHO-K1 cell membranes expressing the hMCHR1. Although no significant difference of the Ki of (±)-SNAP-7941 and (+)-SNAP-7941 was found, the Hill slope factor of (+)-SNAP-7941 was significantly higher, indicating a strong positive cooperativity. Potential advantages and physiological cross-influences due to high positive binding cooperativity for the *in vivo* assessment of the MCHR1 pharmacology still remain unclear and need to be evaluated in future imaging studies. However, FE@SNAP and (±)-SNAP-7941 evinced no binding cooperativity and compared to the natural hormone MCH, differences were proved to be statistically not significant. It has been described that the MCHR1 is expressed in central brain regions, like the lateral hypothalamus, inceto-hypothalamic area and the zona incerta^1^, but recent studies revealed also a high level of MCHR1 expression in the ependymal cells of the ventricular system^22^. Since there is still a lack of potential imaging biomarkers targeting the MCHR1, the study focused on the potential of [^11^C]SNAP-7941 and [^18^F]FE@SNAP to specifically label MCHR1-rich regions in healthy rats under baseline and displacement conditions. Intravenous injection of [^11^C]SNAP-7941 and [^18^F]FE@SNAP evinced high tracer uptake in the ventricular system (see Figs 3 and 4), which supports the expression of the MCHR1 in the ventricular system as reported in previous studies^22, 23^. Further quantitative analysis depicted a clear and statistically significant reduction of the tracer uptake for both [^11^C]SNAP-7941 and [^18^F]FE@SNAP after displacement with (±)-SNAP-7941, whereas displacement with the vehicle revealed no effect (Fig. 5), indicating high specific radiotracer uptake on MCHR1-rich regions on the ependymal cells of the ventricular system. Compared to the uptake in the ventricular system, whole brain uptake was rather low for both [^11^C]SNAP-7941 and [^18^F]FE@SNAP. Detailed quantitative analysis of the whole brain uptake of [^18^F]FE@SNAP before and after displacement with (±)-SNAP-7941 revealed no significant difference. Interestingly, we found a significant difference in the whole brain uptake before and after displacement with (±)-SNAP-7941 for [^11^C]SNAP-7941 (Fig. 6). The result may be biased due to the higher specific uptake in the ventricular system of [^11^C]SNAP-7941 and the fast metabolism of [^18^F]FE@SNAP^30^. Knowing from initial preclinical experiments with [^11^C]SNAP-7941^31^, the low uptake in MCHR1 rich regions in the brain may be caused by limited blood-brain-barrier penetration due to binding to P-glycoprotein (P-gp) and Breast Cancer Resistance Protein (BCRP).

Since the MCHR1 is highly expressed in the ependymal cells of the epithelium of the ventricular system, a tracer for the MCHR1 should show specific uptake and be significantly reduced by an unlabeled ligand in these areas. The specificity of [^11^C]SNAP-7941 and [^18^F]FE@SNAP was successfully proven in small animal PET studies by displacement with the unlabeled MCHR1 antagonist (±)-SNAP-7941, which confirmed that both radiotracers are highly specific agents for MCHR1 imaging. Further, these results were affirmed by *ex vivo* brain autoradiography in a previous study^32^. Since the MCHR1 is primarily involved in the integrated regulation of energy homeostasis, [^11^C]SNAP-7941 and [^18^F]FE@SNAP may serve as a useful tool for imaging and therapy monitoring of a broad range of connected disease such as diabetes, adiposity and insulin resistance. Given the fact that ependymal cells and MCH neurons are both involved in glucose sensing^24–26^ MCH fibres could control the activity of ciliated cells to initiate an increase in CSF flow to meet metabolic needs. Therefore both [^11^C]SNAP-7941 and [^18^F]FE@SNAP may serve a high potential candidates to investigate the involvement of the MCH-system in non-neuronal intercellular communication. Future small animal experiments will focus on the global pharmacodynamics and –kinetics of both radiotracers addressing the peripheral involvement of MCHR1. These insights should facilitate the monitoring and treatment of MCHR1 related pathologies.

## Methods

### Radioligands and chemical compounds

The radioionated no carrier added (n.c.a.) MCHR1 agonist [^125^I]-Tyr^13^-melanin-concentrating hormone ([^125^I]MCH) was purchased from PerkinElmer® (PerkinElmer, Inc., Waltham, MA, USA). The radioligand [^125^I]MCH was described to reveal high affinity towards the MCHR1^13, 35^ and used to serve as a radiolabelled reference compound for the competitive binding experiments.

The non-radioactive reference compound MCH was purchased from Sigma-Aldrich^®^ (Sigma-Aldrich, St. Louis, MO, USA). The MCHR1 antagonists including the racemic mixture of SNAP-7941 ((±)-SNAP-7941), the enantiomeric form (+)-SNAP-7941 and the fluoroethylated analogue (+)-(2-Fluoroethyl)(4 S)-3-{[(3-{4-[3-(acetylamino)phenyl]-1-piperidinyl}propyl)amin]carbonyl}-4-(3,4-difluorophenyl)-6-(methoxymethyl)-2-oxo-1,2,3,4-tetra-hydro-5-pyrimidenecarboxylate (FE@SNAP), as well as the precursor compounds (4S)-3-{[(3-{4-[3-(acetylamino)phenyl]-1piperidinyl}propyl)amino]carbonyl}-4-(3,4-difluorophenyl)-6-(methoxymethyl)-2-oxo-1,2,3,4-tetra-hydro-5-pyrimidenecarboxylate acid (SNAP-acid) and 2-(Tosyloxy)ethyl-3-{[(3-{4-[3-(acetylamino)phenyl]-1piperidinyl}propyl)amino]carbonyl}-4-(3,4-difluorophenyl)-6-(methoxymethyl)-2-oxo-1,2,3,4-tetra-hydro-5-pyrimidenecarboxylate acid (Tos@SNAP) were synthesized in collaboration with the Department of Pharmaceutical Chemistry and the Department Organic Chemistry of the University of Vienna (Vienna, Austria) as previously reported^36, 37^. All other chemicals were of analytical grade and purchased from commercial sources.

### Tracer preparation

Radiosynthesis of [^11^C]SNAP-7941, the radiolabeled analogue of (±)-SNAP-7941, was performed in a fully automated synthesizer (TRACERlab™ FX C Pro, GE Healthcare, Germany) as previously reported^28^. Radiosynthesis of [^18^F]FE@SNAP was performed in a microfluidic device (Advion NanoTek®, Ithaca, NY, USA) as described elsewhere^29, 30^, followed by a purification in a conventional synthesizer unit (Nuclear Interface®, GE Medical Systems, Uppsala, Sweden). Both radiotracers are formulated in physiological saline solution for intravenous injection. Radiochemical purity and molar activity of [^11^C]SNAP-7941 and [^18^F]FE@SNAP were determined by analytical radio-HPLC (Agilent, Boeblingen, Germany).

### Competitive binding studies

Competitive binding studies were conducted on CHO-K1 cell membranes expressing the hMCHR1 (PerkinElmer, Inc., Waltham, USA). Briefly, cell membranes (10 µg/mL) were dissolved in 500 µL 50 mM Tris buffer (pH 7.4) (containing 10 mM MgCl_2_, 2 mM EDTA, 0.1% bacitracin and 0.2% BSA). The equilibrium inhibition constant (K_i_) was evaluated using several concentrations (0.1–10 000 nM) of MCH, (±)-SNAP-7941, (+)-SNAP-7941 and FE@SNAP in the presence of 0.1 nM [^125^I]MCH. All non-labelled compounds were initially dissolved in DMSO and diluted with deionized water to the final concentration, where the amount of DMSO never exceeded 5%. The membranes were incubated in vials at room temperature for 120 min. Subsequently, bound and free fractions of radioligand were separated by centrifugation at 40 000 × *g* for 20 min. The supernatants were removed into new vials and pellets were washed twice with 800 µL ice cold Tris buffer. The pellets were finally dissolved in 1300 µL Tris buffer and the radioactivity of both, the supernatant and the pellet, was measured in a Gamma Counter (2480 WIZARD^2^, PerkinElmer, Waltham, MA, USA). The half-maximum inhibitory concentration (IC_50_) was determined using GraphPad Prism 6.0 (GraphPad Software, Inc., San Diego, CA, USA) and converted into the K_i_ using the Cheng Prusoff equation^38^.

### Animals

Twelve-weeks old male Sprague-Dawley rats (HIM:OFA, Himberg, Austria) weighing 389 ± 86 g were kept under controlled environmental conditions (22 ± 1 °C; 40–70% humidity; 12 hours light/dark cycle) with free access to water and standard laboratory animal diet (sniff R/M-H, sniff Spezialdiaeten GmbH, Soest, Germany). Prior to each experiment, the animals were placed into an induction chamber and anesthetized with 2.5% isoflurane. When unconscious, the animals were taken from the chamber and kept under anesthesia with 1.5–2.5% isoflurane provided via a mask during the whole experiment. Physiological parameters and the depth of anesthesia were monitored continuously. Administration of radioligands and MCHR1 antagonists were performed intravenously via the lateral tail vein. All procedures and protocols using animals have been approved by the Institutional Animal Care and Use Committee of the Medical University of Vienna, Austria, as well as by the Austrian Ministry of Science, Research and Economy (BMWFW-66.009/0029-WF/V/3b/2015). Every effort was made to minimize both, the suffering and the number of animals. All experimental procedures and protocols used in this study were performed in accordance with the relevant guidelines and regulations.

### Small-animal imaging

Anaesthetized rats were immobilized in a multimodal animal carrier unit (MACU; medres® – medical research GmbH, Cologne, Germany) and maintained at a body temperature of 37 °C throughout the whole experiment. Animals received either [^11^C]SNAP-7941 or [^18^F]FE@SNAP, followed by an injection of (±)-SNAP-7941 (15 mg/kg body weight, freshly dissolved in 400 µL; displacement condition; n = 3) or 400 µL of the respective solvent (vehicle condition; n = 3). The MCHR1 antagonists and the vehicle were administered either 15 min ([^11^C]SNAP-7941) or 20 min ([^18^F]FE@SNAP) after radiotracer injection as a bolus via the lateral tail vein. A stereotactic holder attached to the MACU was used to fix the head of the animals in a reliable and reproducible position within the whole imaging study. Experiments were initiated with a 7 minute cone beam attenuation CT (CBCT) of the brain (360 projections; binning 4 × 4; 80 kV; 500 µA; 200 ms exposure time) using a small-animal CT scanner (Siemens Inveon microSPECT/CT, Siemens Medical Solutions, Knoxville, USA). Subsequently the animals were positioned in the imaging chamber of a Siemens Inveon microPET scanner (Siemens Medical Solution, Knoxville, USA). [^11^C]SNAP-7941 (75.85 ± 5.80 MBq; molar activity: 33.12 ± 25.73 GBq/µmol; radiochemical purity: >99%) or [^18^F]FE@SNAP (44.12 ± 4.41 MBq; molar activity: 22.18 ± 9.72 GBq/µmol; radiochemical purity: >90%) was injected (200–800 µL) via the lateral tail vein and dynamic PET imaging was performed 45 min for the [^11^C]-labeled radiotracer and 60 min for the [^18^F]-labeled ligand. Immediately afterwards, T1-weighted high-resolution axial, coronary and sagittal brain MRI scans (2D FLASH; echo time: 3.85 ms; repetition time: 282 ms; flip angle: 30°; field of view: 35 × 35 mm; resolution: 68 × 68 µm; slice thickness: 0.4 mm) were performed using a Bruker BioSpec 94/30 USR small-animal MR system (Bruker BioSpin GmbH, Karlsruhe, Germany). At the end of the imaging study animals were sacrificed under anesthesia through an intravenous injection of pentobarbital sodium (Release® 300 mg/mL, WDT, Garbsen, Germany), brains were removed, weighed and subjected to radioactivity measurements in a Gamma Counter (2480 WIZARD^2^, PerkinElmer, Waltham, MA, USA). Values were normalized to weight and dose and expressed as the percentage injected dose per gram of tissue (%ID/g).

### Image reconstruction and data post processing

CT raw data was reconstructed with a Feldkamp algorithm using a Shepp-Logan filter followed by standard rat beam-hardening correction and noise reduction (matrix size: 1024 × 1024; effective pixel size: 97.56 µm). PET list mode data was sorted into three-dimensional sinograms according to the following frame sequences, for ﻿[^11^C]SNAP-7941: 1 × 3 s, 3 × 2 s, 1 × 6 s, 1 × 15 s, 1 × 35 s, 1 × 145 s, 1 × 270 s, 1 × 285 s, 1 × 165 s, 3 × 30 s, 1 × 120 s, 1 × 240 s, 1 × 420 s, 1 × 900 s and for [^18^F] FE@SNAP: 1 × 3 s, 3 × 2 s, 1 × 6 s, 1 × 15 s, 1 × 35 s, 1 × 145 s, 2 × 270 s, 1 × 285 s, 1 × 165 s, 3 × 30 s, 1 × 120 s, 1 × 240 s, 2 × 487 s, 1 × 976 s. PET images were reconstructed using an OSEM 3D/OP-MAP scatter corrected reconstruction algorithm and a ramp filter (matrix size 128 × 128). The data was normalized and corrected for random, dead time and radioactive decay. A calibration factor was applied to the data for converting units of the microPET images into absolute radioactivity concentration units.

Multimodal (microPET/CT/MRI) rigid-body image registration and biomedical image quantification was performed using the image analysis software PMOD 3.8 (PMOD Technologies Ltd, Zurich, Switzerland) and Inveon Research Workplace (IRW; Siemens Medical Solutions, Knoxville, USA). Volumes of interest (VOIs), comprising the whole brain and the ventricular system of the rats, were outlined on multiple planes of the CT and MRI images and transferred to the PET images of the individual time frames. Time–activity curves (TACs) were calculated, normalized to dose and weight and expressed as standardized uptake values (SUV; g/mL) to facilitate comparison.

### Statistical analysis

Unless mentioned otherwise all experimental data are expressed as mean ± SEM from at least three independent experiments with different batches of radioligand. Statistical testing was performed using GraphPad Prism 6 (GraphPad Software, Inc., San Diego, CA). Descriptive statistical measures were used to confirm the goodness of the nonlinear regression models. Differences among groups and conditions were proved using either a two tailed, unpaired Student’s *t*-test with Welch’s correction or a two-tailed parametric paired *t*-test. Multiple comparisons testing were performed using either ordinary one-way ANOVA with Tukey’s correction or ordinary two-way ANOVA with Sidak’s correction. Values of *P* < 0.05 were considered as statistically significant.
